# Scalable Preparation and Improved Discharge Properties of FeS_2_@CoS_2_ Cathode Materials for High-Temperature Thermal Battery

**DOI:** 10.3390/nano12081360

**Published:** 2022-04-15

**Authors:** Qianqiu Tian, Jing Hu, Shiyu Zhang, Xiaopeng Han, Hao Guo, Licheng Tang, Jiajun Wang, Wenbin Hu

**Affiliations:** 1 School of Materials Science and Engineering, Tianjin University, Tianjin 300072, China; tianqianqiu@tju.edu.cn (Q.T.); zsy272@tju.edu.cn (S.Z.); wangjjtju@126.com (J.W.); wbhu@tju.edu.cn (W.H.); 2 Shandong Engineering Research Center of Green and High-Value Marine Fine Chemical, Weifang University of Science and Technology, Shouguang 262700, China; 3 Haihe Laboratory of Sustainable Chemical Transformations, Nanjing University, Tianjin 200192, China; 4 State Key Laboratory of Advanced Chemical Power Sources, Zunyi 563003, China; guohao_powersources@outlook.com (H.G.); tcl19851221@163.com (L.T.)

**Keywords:** thermal batteries, FeS_2_@CoS_2_ cathode, high specific energy, core–shell structure

## Abstract

Long-time thermal batteries with high specific energy are crucial for improving the fast response ability of long-range weapons. Due to its high capacity, safety, and stability, the new sulfide cathode has attracted extensive attention. In this study, an FeS_2_@CoS_2_ composite cathode with a core–shell structure was prepared via a combination of hydrothermal and high-temperature vulcanization processes. The novel FeS_2_@CoS_2_ cathode not only delivers a high discharge voltage and output capacity, but also has high thermal stability and excellent conductivity. Benefiting from the synergistic effect of FeS_2_ and CoS_2_, the as-synthesized cathode yields a high specific capacity. At a large current density of 1 A/cm^2^, the utilization rate of FeS_2_@CoS_2_ cathode material can reach 72.33%, which is 8.23% higher than that of FeS_2_. Moreover, the maximum output capacity is up to 902 As/g, with a utilization rate of 79.02% at 500 mA/cm^2^. This novel design strategy holds great promise for the development and application of high-performance thermal batteries in the future.

## 1. Introduction

The thermal battery is a type of high-temperature battery with solid molten salt as the electrolyte [1,2,3]. When in use, the internal fireworks source heats the battery stack to the working temperature for discharge [1]. Thermal batteries have the advantages of a high power density, strong environmental adaptability, long storage life, and easy installation and maintenance and they are widely applied in weapons and equipment systems [4], such as strategic and tactical missiles, torpedoes, guided bombs, nuclear weapons, and other emergency fields [5,6]. Due to the separation from a ground power supply, the system power supply of long-range weapons in flight mainly depends on thermal batteries. At the same time, the operation of high-power electric servo mechanism systems demands a persistent high current for a long period of time to achieve posture adjustment or steering of the equipment. For example, the steering gear system often requires a continuous high current of 1 A/cm^2^, or even a pulse high current of 5 A/cm^2^, which represents a significant demand on the output capacity and internal resistance of the cathode material [7].

As a semiconductor, transition sulfides possess good conductive properties and are relatively ideal cathode materials for thermal batteries [8,9]. Among them, natural pyrite FeS_2_, with its high discharge voltage (about 2.1 V), high theoretical capacity (1206 As/g), stable performance, and low price, is a mature cathode material for industrial applications [9]. However, FeS_2_ will violently decompose into Fe7S_8_ and S_2_ at 550 °C, resulting in low output capacity [6,10]. Additionally, the decomposed sulfur vapor produces a severe thermal effect with the anode and other substances in the batteries, and short-circuit combustion can occur in serious cases. By comparison, the synthetic pyrite type CoS_2_ cathode material, with high thermal stability (decomposition temperature of about 650 °C) [11,12,13], high conductivity, and low polarization under a high current density, is considered an ideal alternative to FeS_2_. CoS_2_ material has many defects, such as a relatively low discharge voltage (about 2.0 V) and theoretical capacity (1045 As/g), artificial synthesis, and a high price [8]. Hence, the development of cathode materials with a low cost and specific characteristics for thermal batteries has attracted extensive interest among scholars [14,15]. As a cathode material, NiCl_2_ has a high power density [4,16,17,18], but easily dissolves in halogen salt electrolyte, leading to a short working time and slow activation speed [19,20]. The performance of the CoS_2_ surface is improved by C coating, but this requires a complex production process [12,21]. The new cathode (MnO_2_, Cu_2_O) can only be applied at a low current density and does not tolerate a high current environment [22,23].

Herein, we designed an FeS_2_@CoS_2_ material with a core–shell structure as a cathode for thermal batteries. Combining the advantages of FeS_2_ and CoS_2_, the as-synthesized FeS_2_@CoS_2_ delivered a superior electrochemical performance. The utilization rate of the FeS_2_@CoS_2_ cathode material can reach 72.33% at a large current density of 1 A/cm^2^. Moreover, the specific capacity can reach 902 As/g at 500 mA/cm^2^, realizing a high-capacity output. This study has significance for the further research of high-performance sulfide materials, and has great practical value for the development of high specific energy long-time thermal batteries.

## 2. Material and Methods

### 2.1. Materials

The raw chemicals and solvents used in the experiment were analytical reagents without further purification: Cobalt Nitrate (Co(NO_3_)2·6H_2_O, Sigma-Aldrich LLC, Shanghai, China), Sodium thiosulfate (Na_2_S_2_O_3_·5H_2_O, Sigma-Aldrich LLC.), Iron disulfide (FeS_2_, Sigma-Aldrich LLC.), Sulfur (S, Sinopharm Chemical Reagent Co., Ltd., Shanghai, China).

### 2.2. Preparation of the FeS_2_@CoS_2_ Composite Cathode

Typically, 3.04 g of Co(NO_3_)_2_·6H_2_O and 5.19 g of Na_2_S_2_O_3_·5H_2_O were added to 50 mL deionized water with ultrasonic vibration. FeS_2_ was weighed according to the molar ratio of Co and Fe (Co:Fe = 1:9; 2:8; 3:7; 4:6), and added into the above solution. The resulting solution was transferred to a 100 mL Teflon-lined stainless-steel autoclave. Afterwards, the sealed autoclave was placed in the oven for the hydrothermal reaction at 180 °C for 4 h. Then, the cooled product was filtered, washed several times with deionized water, and dried in vacuum oven at 80 °C for 6 h.

The dried precursor was fully mixed with enough sulfur powder, and calcined in N_2_ (50 sccm) at 550 °C for 6 h with a heating rate of 10 °C/min in a tube furnace. After the reaction, the excess sulfur was removed at 480 °C for 4 h to keep it loose and porous. After cooling to room temperature, the final product was taken out to obtain the core–shell structure FeS_2_@CoS_2_ composite cathode material. According to the contents of Fe and Co in the samples, the samples were marked as FeS_2_@10%CoS_2_, FeS_2_@20%CoS_2_, FeS_2_@30%CoS_2_, and FeS_2_@40%CoS_2_.

### 2.3. Preparation and Discharge Test of the Thermal Battery

The single cell was composed of a cathode (FeS_2_@CoS_2_, LiCl-KCl at a weight ratio of 80:20), Li-Si alloy anode (42 mm diameter), and molten salt electrolyte (mixture of 60 wt% MgO binder and 40 wt% LiCl-KCl eutectic). To compare the properties of the composites, the sulfides used in the cathode were natural pyrite FeS_2_, commercial CoS_2_, and prepared FeS_2_@CoS_2_.

The single cells were fabricated by the traditional pressed-powder process. The cathode, electrolyte, and anode materials were pressed into thin slices with a diameter of 42 mm at a pressure of 380 kN, and then the heating powder (Fe/KClO_4_ = 84/16) was made into thin slices with a diameter of 42.5 mm at a pressure of 270 kN.

Discharge test conditions: the unit batteries were discharged at a background current density of 1 A/cm^2^, with pulse current density of 5 A/cm^2^, for 200 ms and four times at 50 s.

### 2.4. Material Characterization

The thermal stability of the as-prepared samples was characterized by thermogravimetry (TG) and differential scanning calorimetry (DSC) using a STA200RV instrument, with a heating rate of 10 °C/min in an N_2_ atmosphere. Field emission scanning electron microscopy (FE-SEM, S4800, Hitachi, Tokyo, Japan, 30 kV) and transmission electron microscopy (TEM, JEOL JEM-2100F, Tokyo, Japan, 200 kV) were used to investigate the structure and surface morphology of the samples. The phase and crystal structures of the samples were determined by X-ray diffraction (XRD, Bruker D8 Advanced, Bruker, Germany) analysis with Cu Kα radiation (λ = 0.15406 nm).

## 3. Results and Discussion

**Figure 1:**
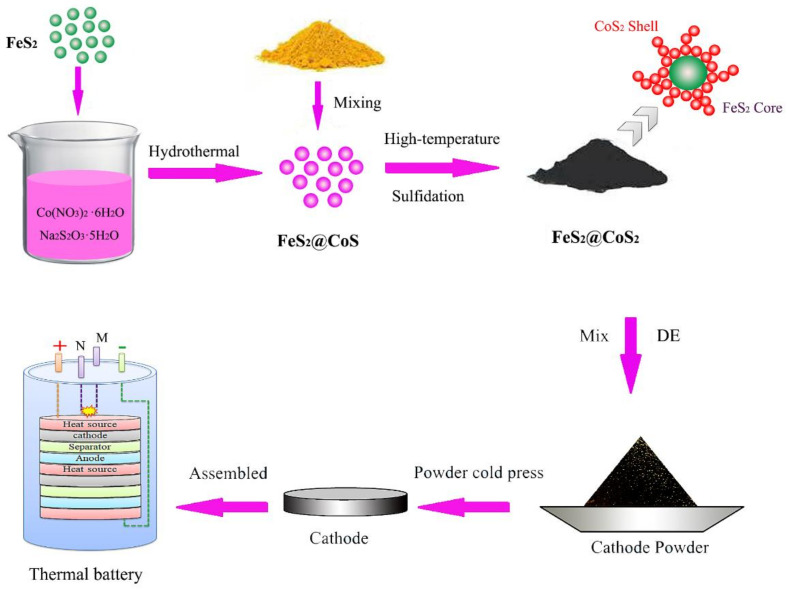
Schematics showing the synthesis process of the FeS_2_@CoS_2_ and battery assembly.

As depicted in Figure 1, the FeS_2_@CoS_2_ cathode material was prepared by hydrothermal and high-temperature sulfidation. Then, the FeS_2_@CoS_2_ cathode, electrolyte, and anode materials were assembled into a single cell by the pressed-powder process. The electrochemical performance test was carried out by connecting single cells in series into unit batteries using special equipment, as shown in Figure S1 (Supplementary Materials).

**Figure 2:**
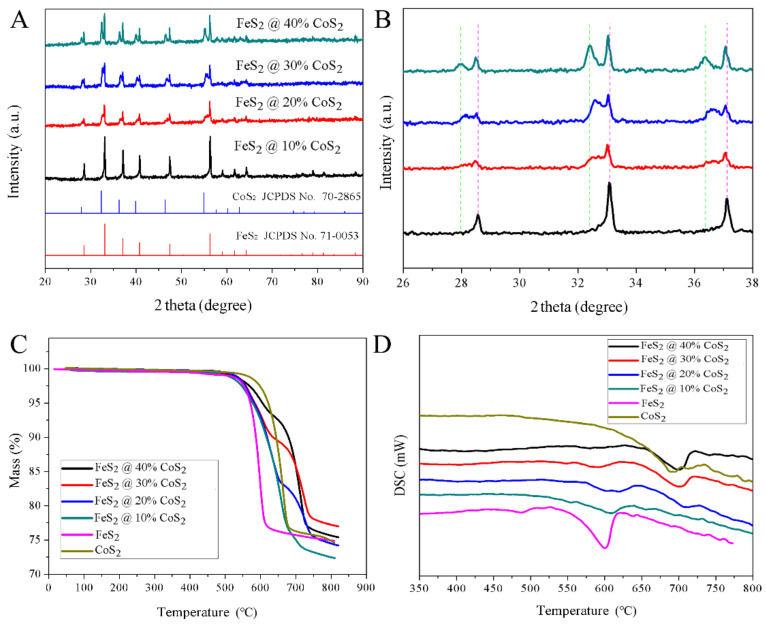
(**A**,**B**) XRD patterns, (**C**) TG and (**D**) DSC curves of FeS_2_@CoS_2_ samples.

Figure 2 displays the X-ray diffraction (XRD) patterns and thermal properties of prepared FeS_2_@CoS_2_ with different CoS_2_ contents. As can be seen from Figure 2A, both FeS_2_ and CoS_2_ belong to pyrite sulfide, and their characteristic diffraction peaks are very similar. Compared with FeS_2_, the diffraction peaks of the CoS_2_ sample appear at a lower angle. The crystallinity of the synthetic materials is high without other diffraction peaks of sulfide in the XRD patterns, indicating that the pyrite type disulfide pure phase is successfully generated via a high-temperature sulfidation process (Figure S2). In addition, the diffraction peaks of the FeS_2_@CoS_2_ composite materials are composed of FeS_2_ and CoS_2_, indicating that the main phases of the composite cathode are FeS_2_ and CoS_2_. As shown in the enlarged XRD patterns of FeS_2_@CoS_2_, the characteristic peaks of FeS_2_ shift to a low angle, while the characteristic peaks of CoS_2_ shift to a high angle. According to the Bragg equation (2dsin θ = *n*λ), if the wavelength of incident light λ is constant, the crystal plane spacing of FeS_2_ increases, while that of CoS_2_ decreases. The above analysis results demonstrate that the introduction of CoS_2_ to FeS_2_ is not a simple physical combination, but leads to distortion of the FeS_2_ lattice.

As shown in Figure 2C, the decomposition temperatures of CoS_2_ and FeS_2_ are 624 and 550 °C, respectively. Moreover, the two single-phase sulfides appear to have only one weight loss step, originating from the sulfur vapor generated by decomposition. The decomposition reaction formula is as follows [8,9]:

(1 − *x*) FeS_2_(s) → Fe_1__− *x*_S(s) (*x* = 0 − 0.2) + 1/2 (1 − 2*x*) S_2_(g) (1)

CoS_2_ → 1/3Co_3_S_4_ + 1/3S_2_(g) (2)

Two obvious weight loss platforms at 545 °C and 680 °C can be observed in the thermogravimetric curves of the FeS_2_@CoS_2_ composites. The first platform is close to the decomposition temperature of FeS_2_. The secondary decomposition is mainly the decomposition of CoS_2_, but is slightly higher than decomposition temperature of CoS_2_, which benefits from the structure of the composite material. At about 550 °C, FeS_2_ decomposes to produce sulfur vapor, which is prevented by the surface coated CoS_2_ and cannot escape. On the thermogravimetric curve, it can be clearly observed that the weight loss rate slows down, and the higher the content of CoS_2_ in the composite material, the slower the weight loss. Meanwhile, the sulfur vapor generated by decomposition forms a sulfur vapor concentration enrichment zone in the material, which will inhibit the decomposition of CoS_2_ from the perspective of thermodynamics, appropriately increasing the decomposition temperature of CoS_2_ to achieve a synergistic effect in the thermal stability of FeS_2_ and CoS_2_. There are two endothermic peaks in the DSC curves (Figure 2D) of all FeS_2_@CoS_2_ samples, corresponding with the thermal decomposition of FeS_2_ and CoS_2_, respectively, which is consistent with the results of thermogravimetric analysis. With the increasing content of CoS_2_ in the sample, the decomposition endothermic peak at 550–650 °C gradually weakens, while the endothermic peak at 650–750 °C becomes stronger. Moreover, no other peaks appear on the differential thermal curve, indicating that the synthesized material is the cobalt iron disulfide phase without another intermediate transition state, which can meet the composition uniformity and stability requirements of a cathode material.

**Figure 3:**
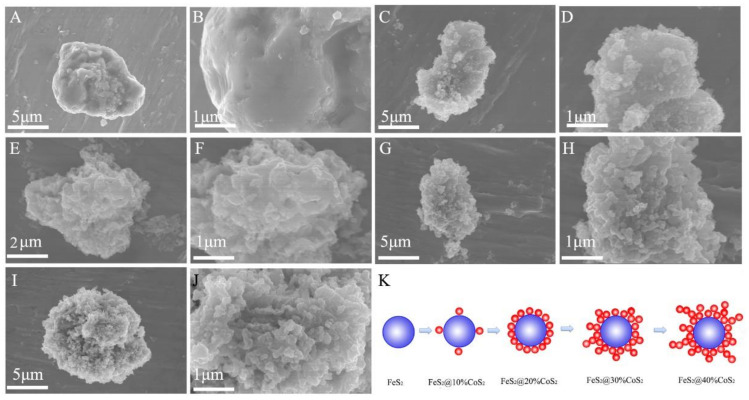
The SEM images of (**A**,**B**) FeS_2_, (**C**,**D**) FeS_2_@10%CoS_2_, (**E**,**F**) FeS_2_@20%CoS_2_, (**G**,**H**) FeS_2_@30%CoS_2_, and (**I**,**J**) FeS_2_@40%CoS_2_. (**K**) Morphology schematic of the FeS_2_@CoS_2_ samples.

To determine the microstructure of the material, sulfides with different cobalt contents were characterized by scanning electron microscopy (SEM). The FeS_2_ core used in the sample synthesis was solid, dense, and smooth, without vertical and horizontal gullies (Figure 3A,B). As shown in Figure 3C–J, as the content of CoS_2_ continuously increases, the coral dendritic structure surface of the product gradually becomes rough, which is consistent with the results after desulfurization (Figure S3). Observations at higher magnification revealed that the dendritic structure can mainly be ascribed to the aggregation and growth of small particles. The special morphology is conducive to increasing the contact area with the electrolyte to enhance the high current loading capacity. Moreover, the coral gully-like structure also contributes to improving the adsorption capacity of the electrolyte, preventing the electrolyte from flowing at high temperatures and achieving greater security of the batteries. Figure 3K shows the morphology schematic of the FeS_2_@CoS_2_ samples. Since FeS_2_ and CoS_2_ are both cubic pyrite structures, and CoS_2_ grows evenly on the surface of FeS_2_, particles with FeS_2_ as the core and CoS_2_ as the shell are formed. Moreover, according to the mapping result of FeS_2_@40%CoS_2_ (Figure S4) and the particle size distribution diagram (Figure S5), the composition of iron, cobalt, and sulfur is relatively uniform, indicating that CoS_2_ and FeS_2_ are well-distributed on a large particle scale.

**Figure 4:**
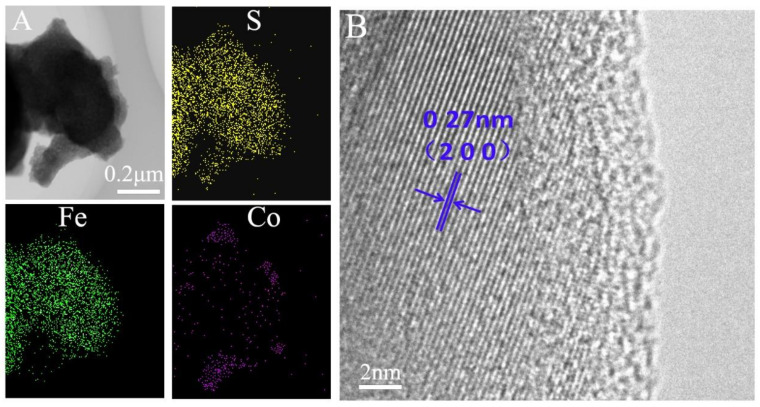
(**A**) Element mappings, (**B**) HRTEM image of FeS_2_@40%CoS_2_.

To further confirm the structure and composition of the samples reliably, transmission electron microscopy (TEM) was also applied to characterize the sample FeS_2_@40%CoS_2_. As illustrated in Figure 4A, the FeS_2_@40%CoS_2_ sample contained iron, cobalt, and sulfur, and iron was mainly concentrated in the core, while cobalt was mainly distributed in the edge area. In addition, the 0.27-nm interlayer distance corresponded to the (2 0 0) crystal plane of CoS_2_ in the HRTEM of FeS_2_@40%CoS_2_ (Figure 4B), indicating that the nanoparticles with a coral dendritic structure are mainly composed of CoS_2_ (Figure S6). Hence, FeS_2_@40%CoS_2_ can be considered as the structure of FeS_2_ as the core and CoS_2_ as the shell, which is conductive to enhancing the thermal stability of the material.

**Figure 5:**
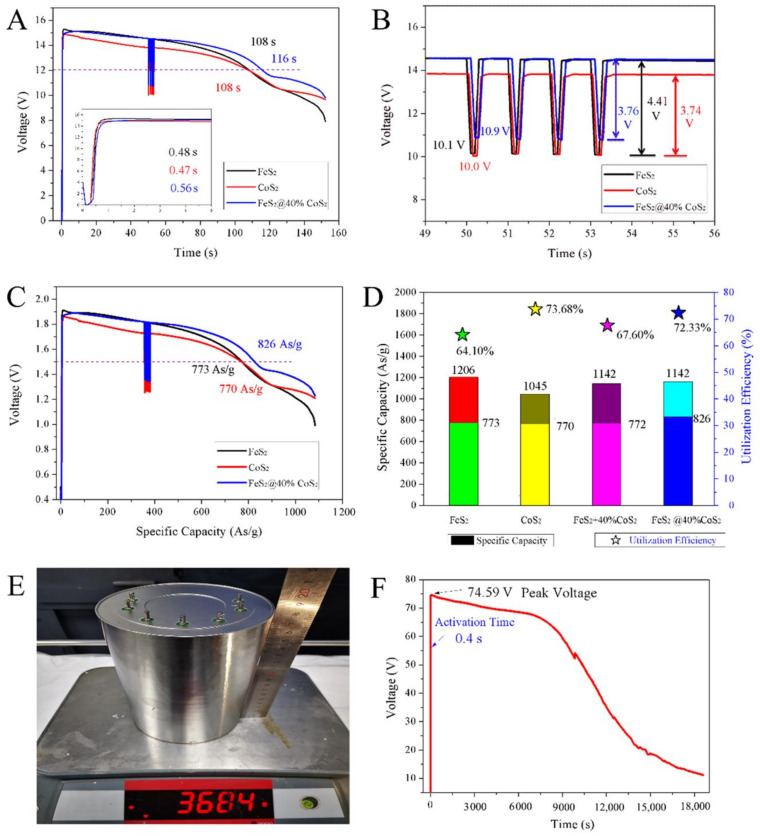
(**A**) The discharge curves of FeS_2_, CoS_2_, and FeS_2_@40%CoS_2_. (**B**) The corresponding changes of the discharge voltage with a pulse current of 5 A/cm^2^. (**C**) The relationship between voltage and specific capacity of FeS_2_, CoS_2_, and FeS_2_@40%CoS_2_ at 1 A/cm^2^. (**D**) The specific capacity and utilization efficiency of FeS_2_, CoS_2_, FeS_2_ + 40%CoS_2_, and FeS_2_@40%CoS_2_. (**E**) Thermal battery sample. (**F**) Safety test curve under a no-load test.

Figure 5 displays the discharge performances of thermal batteries composed of eight single cells with FeS_2_, CoS_2_, and FeS_2_@40%CoS_2_ cathodes. The discharge times of batteries with FeS_2_, CoS_2_, and FeS_2_@40%CoS_2_ at 1 A/cm^2^ were 108 s, 108 s, and 116 s, with the cut-off voltage of 12 V (Figure 5A). By comparison, FeS_2_@40%CoS_2_ exhibits the optimal performance, and the working time is increased by 7.4%. According to the enlarged diagram of activation time, the activation times of FeS_2_, CoS_2_, and FeS_2_@40%CoS_2_ thermal batteries are 0.48 s, 0.47 s, and 0.56 s respectively, which are far below the application requirements of an activation time of 1.5 s, indicating that the prepared FeS_2_@40%CoS_2_ material also possesses the semiconductor properties of sulfide. There is no voltage lag in the activation process, and the voltage can be quickly established, which can satisfy the requirements for rapid activation applications of thermal batteries. Due to the difference in the intrinsic conductivity of materials, the activation time of CoS_2_ was the smallest, followed by FeS_2_. Due to the existence of a two-phase interface and relatively poor crystallinity, FeS_2_@40%CoS_2_ presents a longer activation time.

As shown in the voltage variation diagram under the super current density of 5 A/cm^2^ (Figure 5B), the voltage drop of CoS_2_ thermal batteries is the smallest, at only 3.74 V, but the voltage platform is relatively low, at about 13.8 V. The voltage drops and voltage platform of FeS_2_ thermal batteries are about 4.41 V and 14.6 V, respectively. Obviously, FeS_2_@40%CoS_2_ has both the high voltage platform of FeS_2_ and the low voltage drop of CoS_2_. The voltages of FeS_2_, CoS_2_, and FeS_2_@40%CoS_2_ thermal batteries decreased to 10.1 V, 10 V, and 10.9 V at 5 A/cm^2^. As calculated by Ohm’s law, the internal resistance values of the FeS_2_, CoS_2_, and FeS_2_@40%CoS_2_ thermal batteries were 67.5 mΩ, 79.6 mΩ, and 67.9 mΩ, respectively, so FeS_2_@40%CoS_2_ demonstrates the best high current load performance. 

Figure 5C shows the relationship between voltage and specific capacity of a single cell. As shown, under the current density of 1 A/cm^2^, the peak voltages of FeS_2_, CoS_2_, and FeS_2_@40%CoS_2_ single cells were 1.92 V, 1.86 V, and 1.89 V, respectively, wherein the voltage of FeS_2_@40%CoS_2_ was between FeS_2_ and CoS_2_. Based on the electrical engineering principle, when FeS_2_ and CoS_2_ are used as an external power supply at the same time, due to the voltage difference between them, FeS_2_ will charge to CoS_2_, resulting in the decrease of the potential of FeS_2_ and the increase of the potential of CoS_2_. The specific capacity values of a single cell with FeS_2_, CoS_2_, and FeS_2_@40%CoS_2_ cathodes were 773 As/g, 770 As/g, and 826 As/g, respectively, and the specific capacity of FeS_2_@40%CoS_2_ was increased by 7.3% with the cut-off voltage of 1.5 V.

Figure 5D describes the specific capacity and utilization efficiency of different sulfide cathodes. The specific capacities of FeS_2_ and CoS_2_ were 1206 As/g and 1045 As/g. At 1 A/cm^2^, the utilization rate of the FeS_2_ cathode was 64.10%, while that of the CoS_2_ cathode was 73.68%. According to the ratio of FeS_2_ and CoS_2_ in the composite, the theoretical capacity and utilization rate of 60%FeS_2_ + 40%CoS_2_ were 1142 As/g and 67.6%. However, the actual output specific capacity and utilization rate of the sample were as high as 826 As/g and 72.33%, revealing that there is an obvious synergistic effect in the FeS_2_@40%CoS_2_ cathode. The synergistic effect can be explained as follows: First, compared with FeS_2_, CoS_2_ has higher thermal stability, which weakens the thermal decomposition at high temperatures and improves the effective utilization rate of the cathode material. Second, FeS_2_ itself has a high voltage platform and large theoretical capacity, based on an improved utilization rate, delivering a higher capacity. Third, the conductivity of FeS_2_@40%CoS_2_ is greatly improved compared with that of FeS_2_, characterizing a lower internal resistance and stronger power output capacity.

This synthetic method can be extended to a large number of FeS_2_@CoS_2_ hybrids with over ten grams scale (Figure S7). Moreover, to explore the safety, large-scale (Φ110 × 180) thermal batteries (Figure 5E) were assembled made up of 36 single cells with an FeS_2_@40%CoS_2_ cathode. As depicted in Figure 5F, the voltage curve was gentle and smooth, without a rapid voltage drop or sharp fluctuation in 18,000 s. The no-load result indicated that there is no short circuit or combustion of the thermal battery, revealing no serious decomposition or heating side reaction of the FeS_2_@40%CoS_2_ cathode.

**Figure 6:**
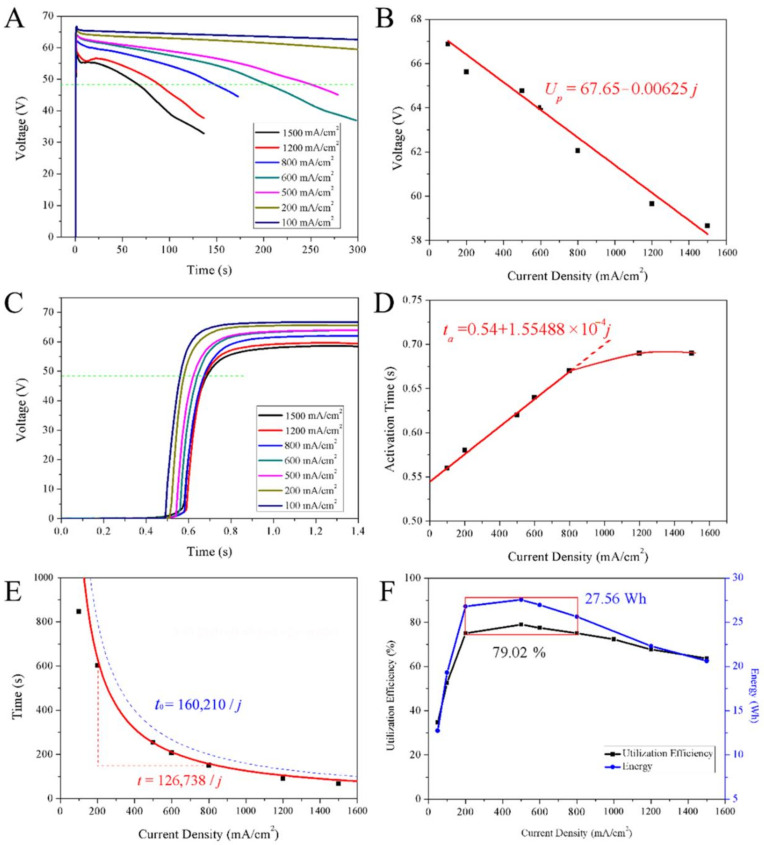
(**A**) The discharge curves of FeS_2_@40%CoS_2_ at different current densities. (**B**) The fitting diagram of peak voltage with current density. (**C**) Activation process of FeS_2_@40%CoS_2_ at different current densities. (**D**) The fitting diagram of activation time with current density. (**E**) The fitting diagram of work time with current density. (**F**) The curves of utilization efficiency and energy with current density.

The discharge curves of thermal batteries composed of 33 single cells with FeS_2_@40%CoS_2_ cathodes are depicted in Figure 6. At a low current density of 200 mA/cm^2^, the discharge voltage was very stable. When the current density was more than 500 mA/cm^2^, the voltage decreased gradually with a large slope. When the current density exceeded 1200 mA/cm^2^, the voltage may be difficult to load and form a concave peak, as the electrolyte of thermal batteries is a high-temperature molten salt with high conductivity, low internal resistance, and a fast discharge speed. The change of the discharge depth and discharge time will lead to the change of the internal resistance of the batteries. Therefore, in the case of high-speed discharge, the voltage decreases with time in a certain slope.

To prevent a too high peak voltage, the desulfurization process adopted in this study effectively reduced the influence of sulfur impurities. It can be seen from Figure 6B that the peak voltage decreased linearly with the increase of current density. Based on Ohm’s law *E* = *U* + *IR_i_* or *U* = *E* − *IR_i_*, when the battery design parameters remain unchanged, the electromotive force *E* and internal resistance *R_i_* are constant values after activation. The current density *j* is positively proportional to the current *I*, and the current is in a linear relationship with the open circuit voltage. By fitting, the linear relationship is *U* = 67.65 − 0.00625*j*. According to the fitting formula, the peak voltage of no-load (*I* = 0 A) is 67.65 V, the voltage of LiSi-FeS_2_@40%CoS_2_ single cell is 2.05 V, and the average internal resistance during activation is about 86 mΩ, suggesting that the internal resistance of the batteries is large at the initial stage of activation.

The activation curves of thermal batteries are smooth ∫ curves (Figure 6C). This manifestation is mainly related to the melting process of molten salt and the change of conductivity. The electrical conductivity of molten salt has a complex exponential relationship with temperature (*κ* = *A*e^(*−Eκ/RT*)^), and the electrical conductivity *κ* is related to factor *A* and activation energy *E_κ_* [24]. At the initial stage of activation, the melting process of molten salt is the main control step, and the open circuit voltage *U* of the batteries is proportional to −*j* e^(*Eκ*/*RT*)^. Therefore, the electrolyte is in a solid state with a voltage of 0 V in activation. When the temperature rises to near the melting point, the molten salt temperature remains stable for a short time due to the phase transformation (Figure S8) [24], and the battery conductivity builds a stable voltage with an exponential relationship. After the phase transformation, the temperature of the molten salt will rise rapidly, and the conductivity will change in an exponential relationship of temperature strengthening, which is manifested as rapidly increasing open circuit voltage. The heating material inside the battery continues to heat the system, so that the temperature of the battery gradually rises and tends to be stabilized, and the open-circuit voltage slowly transitions in an exponential relationship until it becomes stationary. Therefore, the activation process of the battery shows a four-stage variation rule. In the process of establishing voltage in the phase transformation stage of molten salt [25], the current density has the greatest influence on the activation time (Figure 6C). 

As seen in Figure 6D, the activation time of the batteries has a linear relationship with the current density in the early stage, and gradually tends towards constant in the later stage. The linear relationship can be fitted as *t_a_* = 0.54 + 1.55488 × 10^−4^*j*, and the activation time *t_a_* of the batteries is 0.54 s without load (*j* = 0 mA/cm^2^). According to *U*∝−*j* e^(*Eκ*/*RT*)^, under the premise of a loading large current, e^(*Eκ*/*RT*)^ is a constant value at the same time, and the open circuit voltage is linear with the current density *j*. As the current density *j* increases, the open circuit voltage is negatively correlated, so the time for the voltage to reach the active voltage is delayed. When the time is long enough, the molten salt completes the phase transformation and starts to heat up rapidly. Even if the current density increases, the activation time will not be affected, and the activation gradually tends towards becoming stable.

Figure 6E shows the influence of current density on working time. The current density is inversely proportional to the working time of the batteries, with the cut-off voltage of 48 V. After data fitting, the relationship between the two can be expressed as *t* = 126,738/*j*. Calculated with the theoretical specific capacity of FeS_2_@40%CoS_2_ at 1141.6 As/g, the relationship between current density and working time is *t* = 160,210/*j* (blue dotted line). After analysis, within the current density range of 200–800 mA/cm^2^, the working time is consistent with the red fitting line, and the current density is too high or too low, which deviates from the fitting line.

As shown in Figure 6F, the utilization rate and output energy of FeS_2_@40%CoS_2_ increased sharply first and then decreased slowly with increasing current density. In the red box of Figure 6F, when the current density is in the range of 200–800 mA/cm^2^, the maximum output capacity of FeS_2_@40%CoS_2_ is up to 902 As/g, with a utilization rate of 79.02%, and the maximum energy output is up to 27.56 Wh at 500 mA/cm^2^. When the current density is less than 200 mA/cm^2^, the electrode material utilization rate decreases sharply, which can be attributed to the long working time. As molten salt batteries, thermal batteries mainly rely on heat to ensure the electrolyte is in the appropriate working temperature zone. Too long a time will lead to heat loss and internal resistance increase, such that some of the FeS_2_@40%CoS_2_ cannot participate in the electrochemical reaction, resulting in capacity loss. When the current density is larger than 800 mA/cm^2^, the polarization resistance increases with the discharge depth, and the FeS_2_@40%CoS_2_ cannot support the high current load, resulting in a reduced capacity output and utilization rate. Hence, 200–800 mA/cm^2^ is the ideal reference current density for thermal battery design, with the highest utilization rate of materials and maximum output energy.

## 4. Conclusions

In this paper, an FeS_2_@CoS_2_ composite cathode material with a core–shell structure was prepared by a hydrothermal and high-temperature sulfurization process. The cathode has the advantages of the high voltage of FeS_2_ and the high thermal stability and conductivity of CoS_2_, which greatly enhances the actual output capacity and current load capability of the material. At a current density of 1 A/cm^2^, the voltage and specific capacity of a single cell with FeS_2_@40%CoS_2_ is 1.89 V and 826 As/g, with an increase of discharge time of 7.4%. When the current density is in the range of 200–800 mA/cm^2^, the effective utilization rate of the FeS_2_@40%CoS_2_ material is higher than 79.02%, and the energy output is up to 27.56 Wh. This study provides a novel design strategy and high-performance sulfide material for the development of high-specific-energy and long-time thermal batteries.

## Data Availability

The data that support the findings of this study are available on request from the corresponding author.

## Supplementary Materials

The following supporting information can be downloaded at: https://www.mdpi.com/article/10.3390/nano12081360/s1, Figure S1: (A) Dedicated device for testing the thermal batteries; (B) The test schematic diagram; (C) The discharge test conditions; Figure S2: Effect of S/Fe mole ratio on product composition; Figure S3: SEM images of (A–B) CoS_2_ containing sulfur and (C–D) desulfurized CoS_2_; Figure S4: Element mappings of FeS_2_@40%CoS_2_; Figure S5: Particle size distribution diagram of FeS_2_@40%CoS_2_; Figure S6: TEM images of (A–B) FeS_2_ and (C–D) commercial CoS_2_; Figure S7: Scalable preparation of FeS_2_@CoS_2_ composite with over ten grams; Figure S8: Schematic diagram of molten salt temperature rise curve.
