# Supplementary material for: Scalable Preparation and Improved Discharge Properties of FeS2@CoS2 Cathode Materials for High-Temperature Thermal Battery
Source: Nanomaterials (Basel). 2022 Apr 15;12(8):1360. doi: 10.3390/nano12081360 (PMC9030623; doi:10.3390/nano12081360)
Supplement: Supplementary file 1 [file nanomaterials-12-01360-s001.zip › nanomaterials-1669181-supplementary.pdf]

nanomaterials

Supplementary Materials

Scalable Preparation and Improved Discharge Properties of $FeS_2@CoS_2$ Cathode Materials for High-Temperature Thermal Battery

Qianqiu Tian [1], Jing Hu [2,*], Shiyu Zhang [1], Xiaopeng Han [1,3,*], Hao Guo [4], Licheng Tang [4], Jiajun Wang [1] and Wenbin Hu [1]

1 School of Materials Science and Engineering, Tianjin University, Tianjin 300072, China; tianqianqiu@tju.edu.cn (Q.T.); zsy272@tju.edu.cn (S.Z.); wangjjtju@126.com (J.W.); wbhu@tju.edu.cn (W.H.)
2 Shandong Engineering Research Center of Green and High-Value Marine Fine Chemical, Weifang University of Science and Technology, Shouguang 262700, China
3 Haihe Laboratory of Sustainable Chemical Transformations, Nanjing University, Tianjin 200192, China
4 State Key Laboratory of Advanced Chemical Power Sources, Zunyi, 563003, China; guohao_powersources@outlook.com (H.G.); tcl19851221@163.com (L.T.)
* Correspondence: wkhujing@wfust.edu.cn (J.H.); xphan@tju.edu.cn (X.H.)

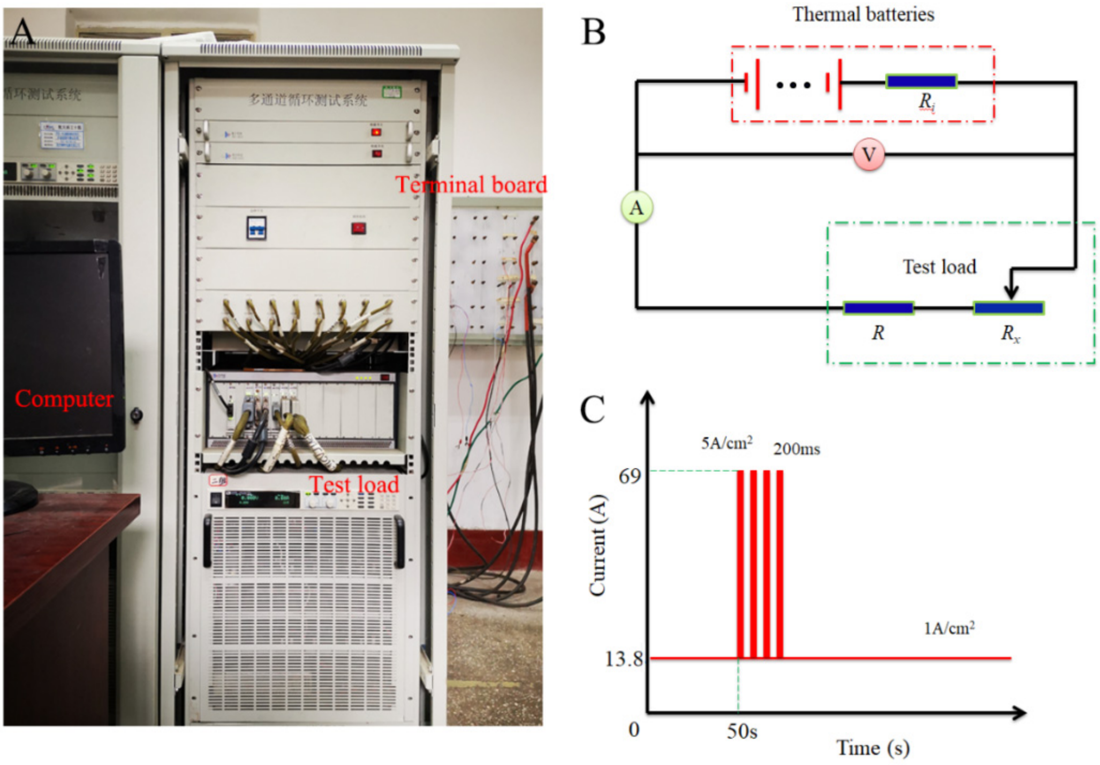

Figure S1. (**A**) Dedicated device for testing the thermal batteries; (**B**) The test schematic diagram; (**C**) The discharge test conditions.

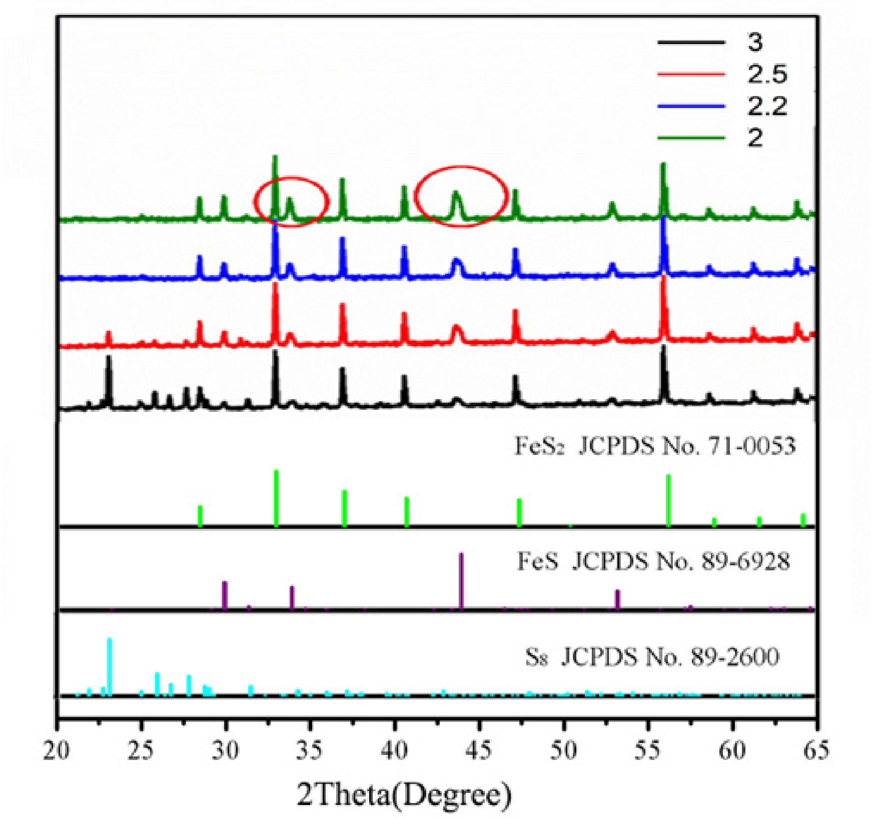

Figure S2. Effect of S/Fe mole ratio on product composition.

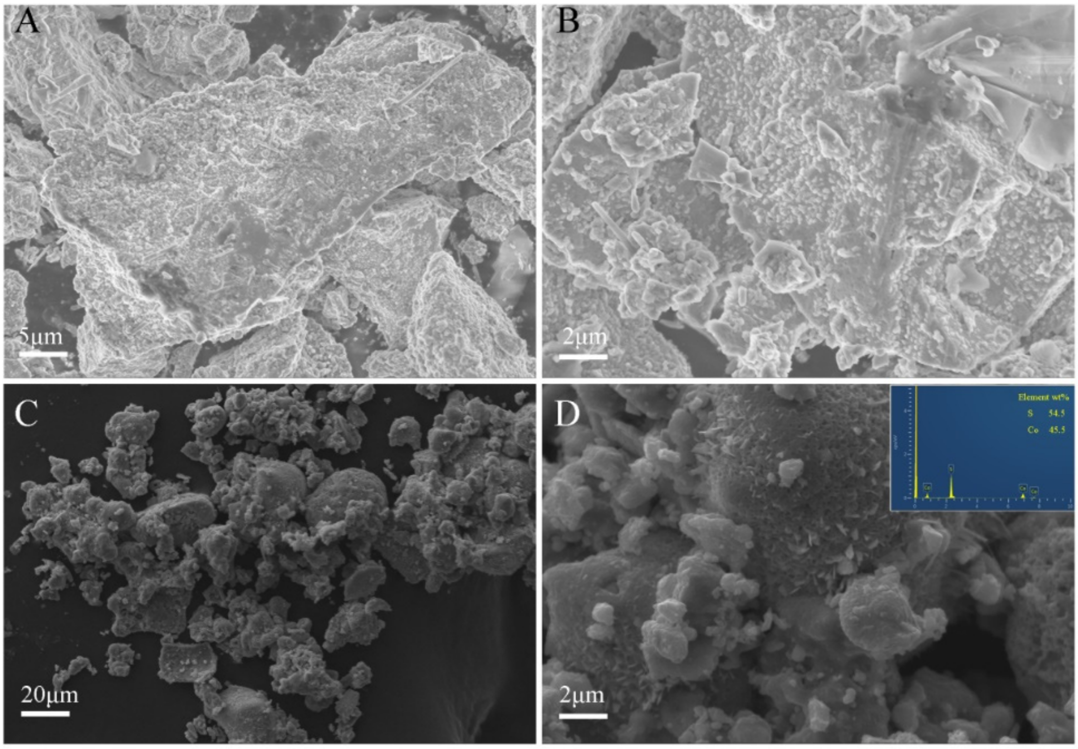

Figure S3. SEM images of (**A–B**) CoS_2 containing sulfur and (**C-D**) desulfurized CoS_2.

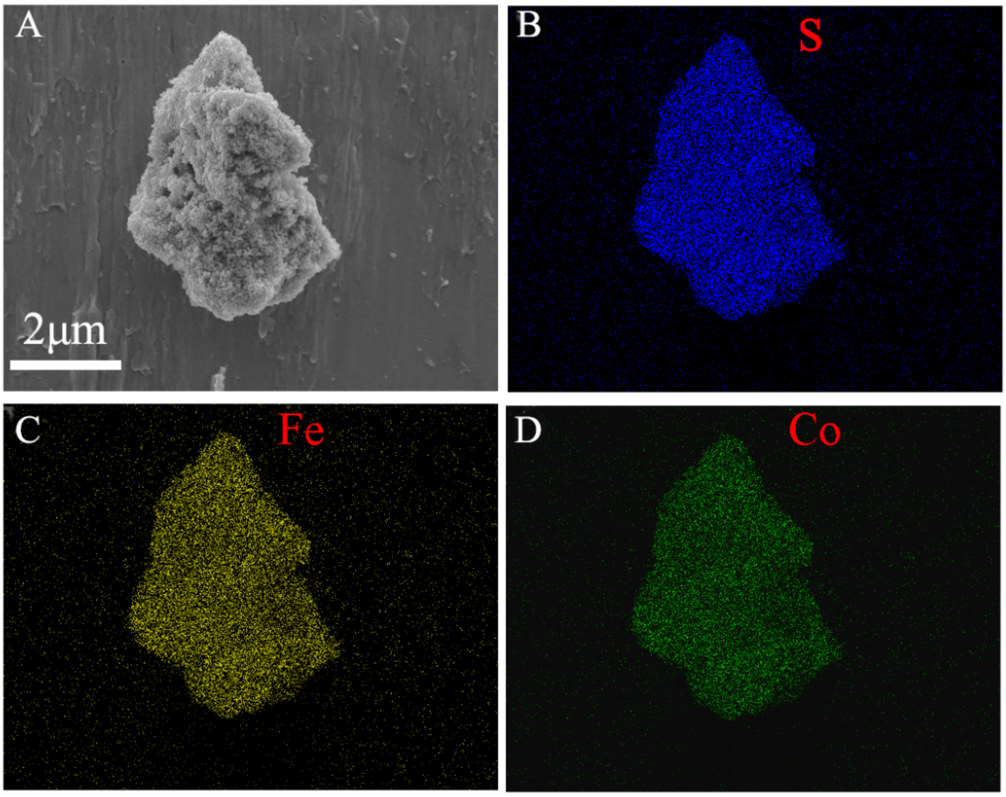

Figure S4. Element mappings of FeS_2@40%CoS_2.

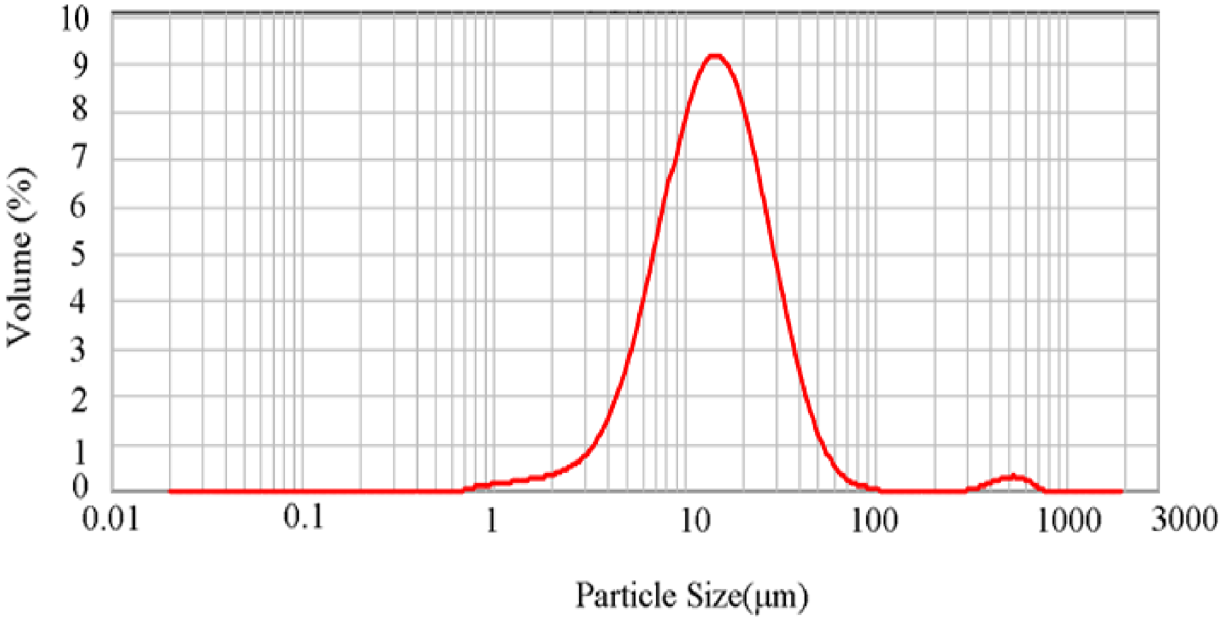

Figure S5. Particle size distribution diagram of FeS_2@40%CoS_2.

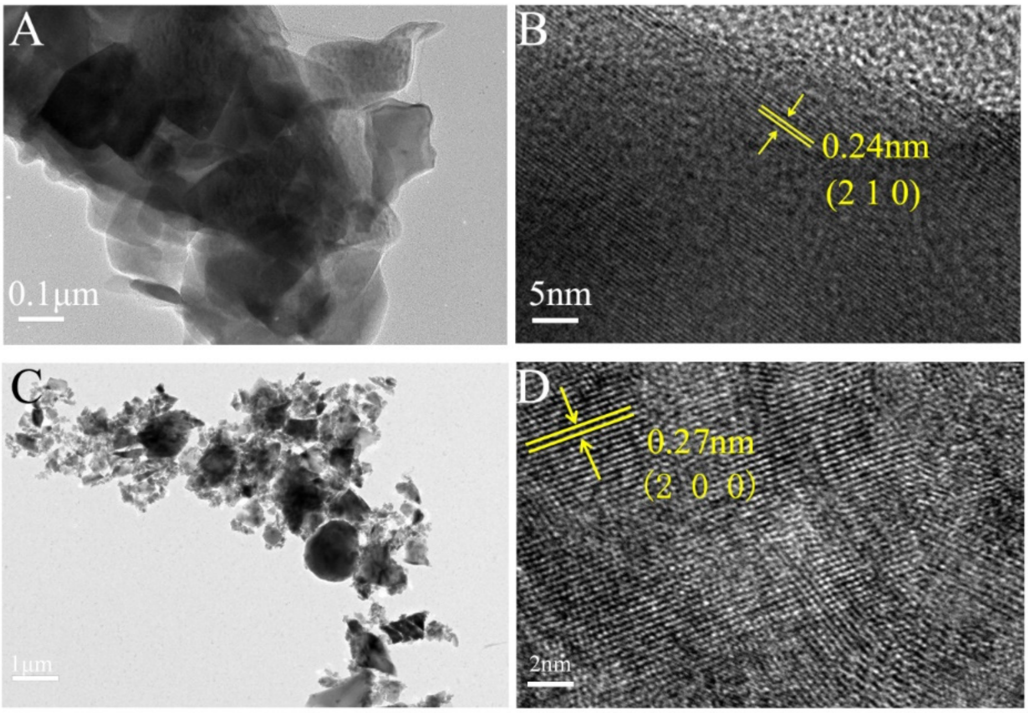

Figure S6. TEM images of (**A–B**) FeS_2 and (**C-D**) commercial CoS_2.

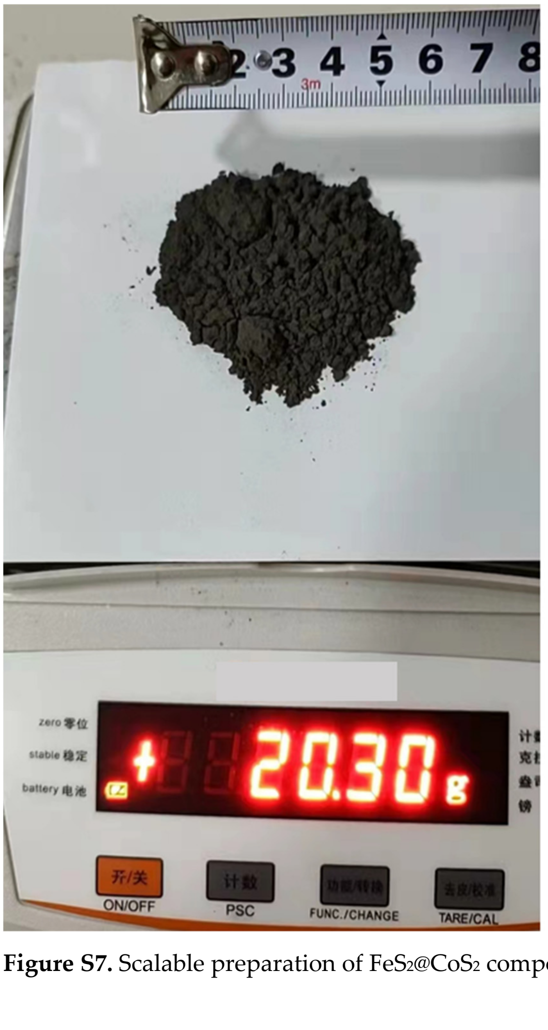

Figure S7. Scalable preparation of $FeS_2@CoS_2$ composite with over ten grams.

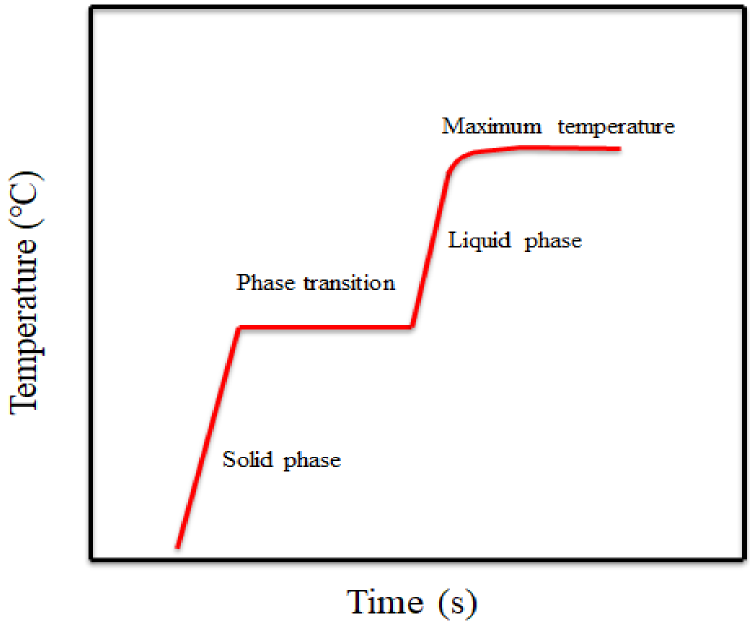

Figure S8. Schematic diagram of molten salt temperature rise curve.
